# CDK12 promotes papillary thyroid cancer progression through regulating the c-myc/β-catenin pathway

**DOI:** 10.7150/jca.42849

**Published:** 2020-04-27

**Authors:** Ning Bai, Fada Xia, Wenlong Wang, Yao Lei, Jiang Bo, Xinying Li

**Affiliations:** Department of General Surgery, Xiangya Hospital, Central South University, Changsha, China.

**Keywords:** CDK12, papillary thyroid cancer, c-myc, β-catenin, cancer progression

## Abstract

**Background**: CDK12 is a potential therapeutic target in papillary thyroid cancer that regulates the c-myc/β-catenin pathway.

**Objective**: We aimed to explore the specific mechanism of CDK12 in papillary thyroid cancer and provide a new target of cancer therapy.

**Methods**: RT-qPCR was used to determine the CDK12 mRNA expression level. An IHC assay was performed to detect the tissue expression of CDK12. Then, we downregulated CDK12 expression in the thyroid cancer cell lines TPC-1-shCDK12 and KAT-5-shCDK12. CCK8 assays, colony formation assays, and animal xenograft models were used to evaluate the effect of CDK12 on tumorigenesis. Transwell assays and *in vivo* metastasis models were used to observe whether CDK12 can promote cancer metastasis. Western blotting further confirmed the mechanism of CDK12 in papillary thyroid cancer through the c-myc/β-catenin pathway.

**Results**: Upregulated CDK12 expression in papillary thyroid cancer promoted papillary thyroid cancer carcinogenesis *in vivo*, and *in vitro* CDK12 strengthened papillary thyroid cancer (PTC) cell migration and tumor metastasis. CDK12 promoted tumor progression by regulating c-myc/β-catenin pathway activation.

**Conclusions**: CDK12 affects the c-myc/β-catenin pathway to stimulate papillary thyroid cancer proliferation and metastasis. Inhibiting CDK12 might be a new method in papillary thyroid cancer therapy.

## Introduction

According to global cancer statistics, thyroid cancer is the fifth fastest growing cancer in the world. A total of 53990 new cases of thyroid cancer were estimated in 2018, the number of female patients will reach 40900 cases, and new cases in men will reach 13090. The incidence of thyroid cancer in women is much higher than in men, and the sex ratio is almost 3:1^1^. Papillary thyroid cancer (PTC) is one subtype of thyroid cancer. PTC incidence has risen rapidly in recent years and mainly induces a high growth rate of thyroid cancer 2.Thyroid cancer pathogenesis may be induced by the unhealthy living habits 3, 4 or environmental factors 5. Although the prognosis of PTC is usually favorable if it is diagnosed in early a stage and is treated well, some patients will still suffer lymph node metastasis, distant metastasis or other vital syndromes. Once metastasis occurs, the prognosis of PTC is poor, and patients will even die 6. Therefore, it is urgent to reveal the detailed mechanism of PTC genesis and metastasis.

CDK12 is a member of the cyclin-dependent kinase (CDK) familythat relies on the regulatory subunit cyclin to phosphorylate the RNA polymerase II C-terminal domain toregulate cell cycle transition. CDK12 generally binds with cyclin K to maintain genomic stability by repairing DNA damage 7. According to reports, CDK12 mutation promotes the progression of multiple cancers, such as breast cancer 8, ovarian cancer 9, and prostate cancer 10. Jerry F. Tien et al analyzed global mRNA transcripts between normal and breast cancer cell lines with and without CDK12 amplification and found that CDK12 primarily regulates alternative last exon (ALE) splicing, which typically regulates DNA damage response activators. CDK12 modulates ALE splicing of the DNA damage response activator ATM and a DNAJB6 isoform that influences cell invasion and tumorigenesis in xenografts 8. The deregulations of CDK12 induces tumorigenesis via DNA repair defects in ovarian cancer, such as inactivation of the homologous recombination (HR) repair pathway 11. Inhibiting CDK12 in cancer cells without CDK12 mutations leads to gene length-dependent elongation defects, inducing cleavage maturation early, polyadenylation and loss of the expression of long (>45 kb) genes 12. Based on the reports on mCRPC, focal tandem duplications (FTDs) associated with CDK12 loss result in highly recurrent gains at loci of genes and marked differential gene expression involved in the cell cycle and DNA replication 13. In summary, knockdown of CDK12/CycK decreases the levels of nascent transcripts of several DDR genes 14, regulates alternative splicing 15 and interferes with effective 3'-end formation of model premRNAs 7. Hee-Joo Choi et al found that high CDK12 expression caused by concurrent amplification ofCDK12 and HER2 in breast cancer patients through activating ErbB-PI3K-AKT or WNT-signaling cascades induced disease recurrence and poor survival 16. This suggests that CDK12 gene amplification contributes to the pathogenesis of cancer. These findings mostly focus on the gene repair pathway of CDK12 in cancer. However, the detailed mechanism of CDK12 in papillary thyroid cancer remains largely unknown.

c-myc is a classical oncogene that can affect the progression of many cancers 17-20. It was determined that c-myc is the downstream target of wnt/β-catenin. c-myc regulates cell proliferation, differentiation, angiogenesis, and apoptosis^21^, ^22^. c-Myc translation is activated by β-catenin. β-cateninis critical in many cancers and participates in a broad range of cancer processes, including stem cell self-renewal, stemness-maintaining progression, and tumorigenesis^23^, ^24^. The c-myc pathway is significantly correlated with poor prognosis, increased possibility of breast-to-lung metastasis and reduced overall survival 25, 26. Herein, we performed this study to investigate the function of CDK12 and its underlying mechanism in papillary thyroid cancer progression. Our data demonstrated that CDK12 promoted cancer proliferation, migration and metastasis through the c-myc/β-catenin pathway. CDK12 may be a potential target in PTC therapy.

## Method and materials

### Clinical sample data

Thirty pairs of clinical samples and adjacent normal tissues were collected from patients who were diagnosed with PTC and underwent surgery at Xiangya Hospital, Central South University from 2015 to 2017, aged 22-60 years old. The sectioned cancerous tissues and paired normal mammary tissues were immediately removed and stored in RNAlater (Ambion), and the RNA was extracted with Trizol (Invitrogen, Carlsbad, CA, USA) and then frozen in liquid nitrogen at -80°C. All patients provided informed consent, and this study was approved by the Medical Ethics Committee of Xiangya Hospital, Central South University.

### Immunohistochemistry

According to the EnVision system (DAKO) instructions, the formalin-fixed and paraffin-embedded sections were dewaxed with xylene and graded ethanol. The sections were microwaved in citrate buffer to retrieve the antigens. Then, the sections were incubated in H_2_O_2_ solution at routine temperature for 15 min. The sections were incubated with primary antibody (CDK12, 1:100, Cell Signaling Technology) overnight at 4°C. The sections were incubated with labeled polymer-HRP anti-mouse at routine temperature for 30 min. The sections were incubated in substrate-chromogen solution for 5-30 min. The slides were dehydrated with xylene and graded ethanol. Images were taken with an optimal microscope, and the results were quantified by two pathologists. The evaluation of IHC progression followed the blind trial rules.

### Cell culture and transfection

PTC cell lines (TPC-1, NPA87 and KAT-5) and the normal cell line Nthy-ori3-1 were from the Chinese Academy of Sciences (Shanghai, China). Cell lines were cultured in DMEM (Gibco, Carlsbad, CA, USA) containing 10% FBS (Gibco) and maintained in a humidified incubator at anatmosphere with 5% CO2. the stable knockdown the CDK12 expression in TPC-1 and KAT-5 cells through using Lenti-Pac™ HIV Expression Packaging Kit (GeneCopoeia) following the manufacturer's instructions.

### Cell Counting Kit-8 Assay

The adherent cancer cells were lysed and 500 sh-CDK12 and sh-CTR cancer cells were seeded into a 96-well plate. The plate was incubated for 24, 48, 72, and 96 h in the incubator. Then, 10 μl of CCK-8 solution was added to each well of the plate. The plate was incubated for 1 - 4 h in the incubator. The absorbance was measured at 450 nm using a microplate reader (Bio-Tek EPOCH2, Vermont, USA).

### Transwell assay

The cells were washed twice with 1x PBS and trypsinized. Then, a volume of 10% FBS in DMEM equal to the volume of trypsin was added to inactivate the trypsin. The cells were gently centrifuged. The cells were resuspended in DMEM and counted. A total of 1 x 10^5^ cells were added into the transwell compartments. And the mitomycin was added in to the culture to avoid the effect of cancer cell's proliferation. The cells were incubated in the transwell plate at 37 °C and 5% CO_2_ for 24 h. The upper layer of cells was swabbed. The lower side of the insert filter was rinsed in4% formaldehyde solution for 10 min. Next, the cells were stained with 1% crystal violet for 15 min. The inserts were washed with PBS 4 times. The insert membrane was dried. The number of cells on the lower side of the filter was counted under a NIKON ECLIPSE 80imicroscope (Nikon Instruments, NY, USA). Each migration condition was tested in triplicate.

### Animal xenograft model

In brief, 1×10^7^sh-CDK12, sh-CTR TPC-1 or KAT-5 cells were subcutaneously injected into the flanks of nude mice from Charles River (Beijing, China). Each group contained 5 nude mice. Twenty-eight days later, the mice were euthanized and then the tumor volumes were analyzed. The animal research was approved by the ethical committee of Xiangya Hospital, Central South University.

### RT-qPCR analysis

qRT-PCR assays were carried out with SYBR Premix Ex Taq™ (Takara, Japan) and an All-in-One™ miRNA qRT-PCR Detection Kit (GeneCopoeia) using a Bio-Rad IQTM5 Multicolor Real-Time PCR Detection System (USA).In brief, total RNA was extracted from PTC tissues and celllines. cDNA was synthesized from 1μg of total RNAin 20 µl reaction volumes using 5x PrimeScript RT Master Mix. PCR amplification was carried out with Taq DNA polymerase using 2µL of the cDNA as a template. The amplification reactions were run with 30 thermocycles of 30s at 94°C, 30 s at 55°C, and 30 s at 72°C. All primers were acquired from Sangon Biotech (Shanghai, China). Forward primer: CDK12: (5'-CTAACAGCAGAGAGCGTCACC-3'), reverse primer: CDK12 (5'-AAAGGTTTGATAACTGTGCCCA-3'). U6 was used as an internal control.

### Western blot analysis

The total protein from cells was isolated with RIPA buffer and PMSF. The proteins were denatured at 100°C for 10 min and then quantified. A total of 20-30 μg of protein was loaded into the wells of the SDS-PAGE gel. The gel was run for 2 h at 100 V. The protein was transferred to the PVDF membrane for 2 h at 200 mA. The membrane was blocked in 5% skimmed milk for 1 h at room temperature. The membrane was incubated with a 1:3000 dilution of primary antibody β-catenin (Santa Cruz Biotechnology), wnt-1 (Abcam), c-myc (Abcam), CD44 (Cell Signaling Technology), slug (Cell Signaling Technology) and snail (Cell Signaling Technology), actin (Abcam) incubate the membrane and primary antibody overnight at 4°C and then exchanged the primary antibody with the secondary antibody at room temperature for 1 h.The protein bands were exposed with a ChemiImager System.

### *In vivo* Metastasis Assay

A total of 1×10^5^ sh-CDK12, sh-CTR TPC-1 or KAT-5 cells were injected into nude mice through the tail vein. 5 nude mice were facilitated for each group. Thirty days later, all mice were anesthetized, and the lungs were extracted. The metastatic nodules were counted by eye and then stored in 4% formaldehyde solution for further study.

### Statistics

All data are reported as the mean ± SD. The paired t-test was used to evaluate the differences of the tumor tissue and paired adjacent normal tissue. The differences between two groups were determined by Student's two-tailed unpaired t-test. A p value <0.05 was regarded as statistically significant.

## Results

### Upregulated CDK12expression in papillary thyroid cancer

**Figure 1:**
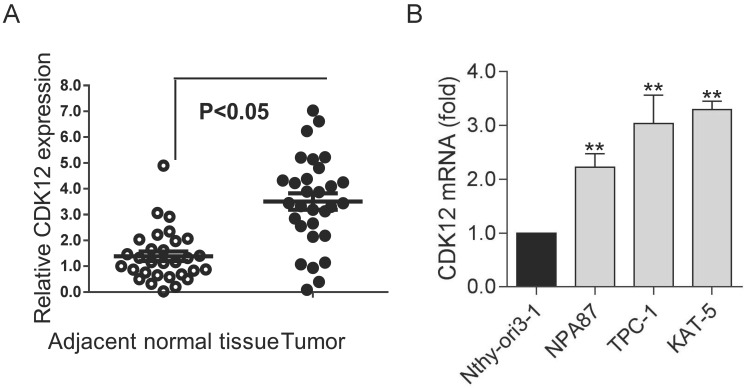
The high expression of CDK12 in PTC: (A) Expression levels of CDK12 in 20 PTC specimens and paired adjacent normal tissues. (B) Expression levels of CDK12 by qRT-PCR in the normal thyroid cell line Nthy-ori3-1 and the TPC-1, NPA87, KAT-5 papillary thyroid cancer cell lines. β-actin was used as the internal control. A P value <0.05 was considered significant. **, P<0.01.

To study the role of CDK12 in PTC, we detected the expression of CDK12 in clinical PTC specimens. We found that CDK12 expression was significantly higher in tumors than in normal tissues (Figure 1A). This result indicated that CDK12 may play an important role in papillary thyroid cancer progression. Then, we detected CDK12 mRNA expression in several common thyroid cell lines. PTC cell lines (TPC-1, NPA87 and KAT-5) had prominently higher CDK12 levels than the normal cell line Nthy-ori3-1. Additionally, the CDK12 mRNA expression levels of TPC-1 and KAT-5 cells were the highest among the papillary thyroid cancer cell lines (Figure 1B). These two cancer cell lines were used for subsequent experiments.

### CDK12 promotes papillary thyroid cancer carcinogenesis *in vivo* and *in vitro*

**Figure 2:**
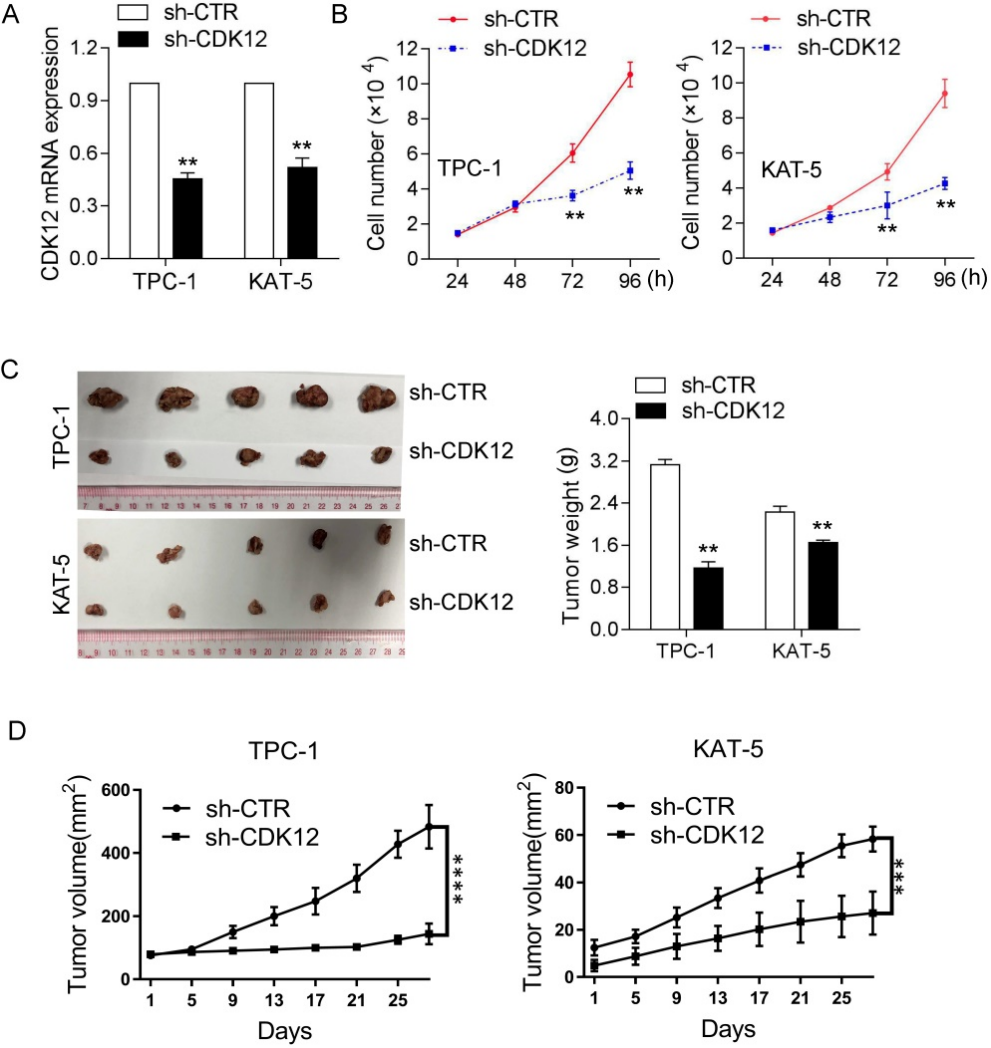
CDK12 promotes PTC progression *in vivo* and *in vitro*. (A): qRT-PCR was used to determine the mRNA expression of CDK12. TPC-1 and KAT-5 cells had effectively decreased mRNA expression of CDK12. (B): A CCK-8 assay was used to detect the cell proliferation ability of TPC-1-sh-CDK12 and KAT-5-sh-CDK12 cells. (C): Representative images of xenografts and the tumor weight statistics in nude mice. The weights of the tumors are shown in the right panel. All results are expressed as the mean±SD of three independent experiments. **, P<0.01. (D): the growth curve of xenografts, TPC-1(left), KAT-5(right), ***, P<0.001, ****, P<0.0001.

To confirm the function of CDK12 in tumorigenicity, we inhibited CDK12 mRNA expression in the TPC-1 and KAT-5 cancer cell lines (Figure 2A). The CCK8 assay results demonstrated that CDK12 downregulation inhibited cancer cell proliferation (Figure 2B). Animal research was performed to further confirm the role of CDK12 carcinogenesis *in vivo*. CDK12 expression was knocked down in the PTC-1 and KAT-5 cell lines and the cells were then injected into nude mice in situ. After 4 weeks, the mice were sacrificed to harvest the tumors, and the tumor volumes were measured. CDK12 inhibition resulted in tumors that were obviously smaller than those in the control group (Figure 2C-D). These results demonstrated that CDK12 promotes papillary thyroid cancer proliferation both *in vivo* and *in vitro*. Hence, CDK12 may play an important role in papillary thyroid cancer progression.

### CDK12 enhancesPTCcell migration and tumor metastasis

**Figure 3:**
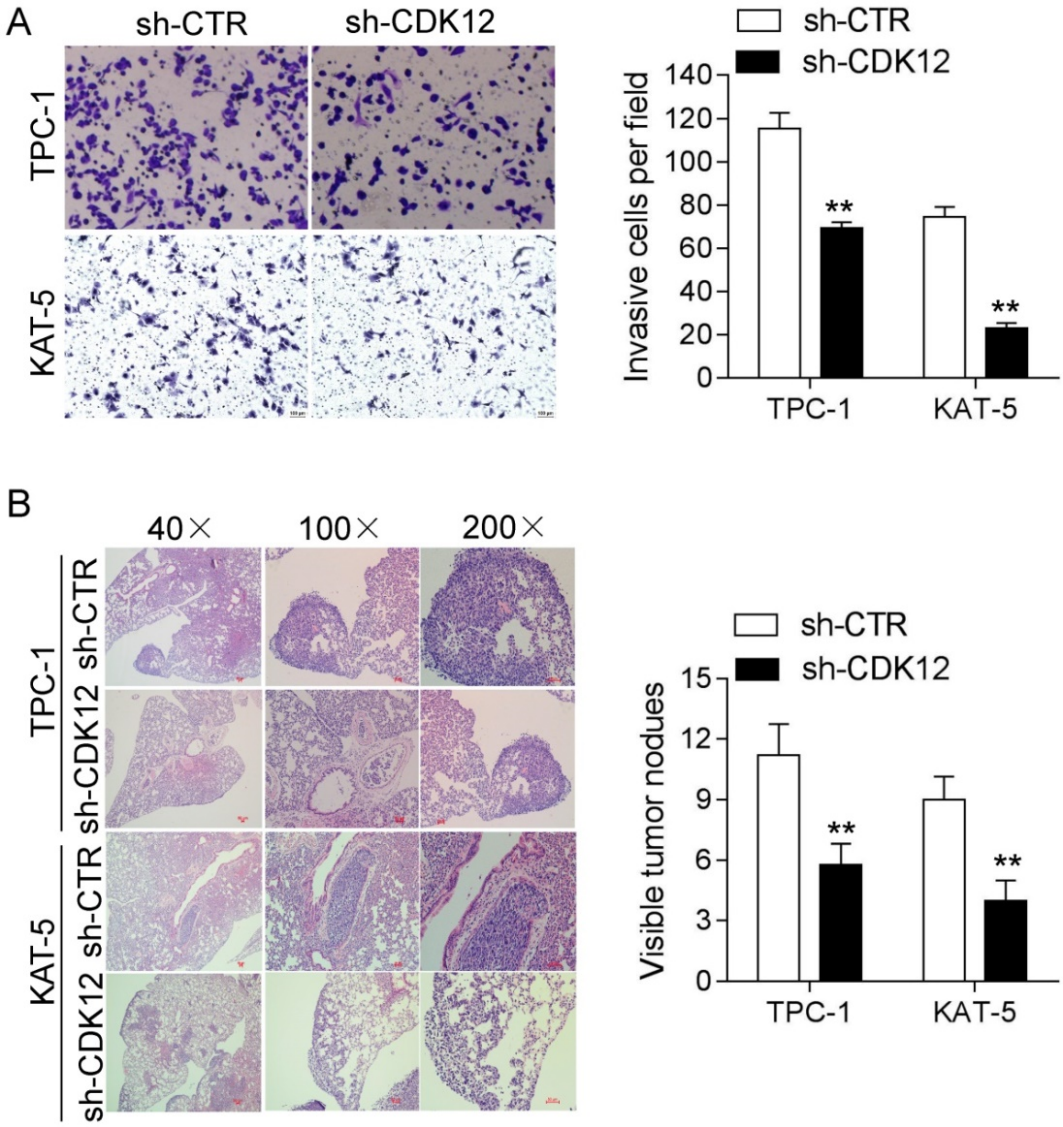
CDK12 promotes PTC cell migration and tumor metastasis. (A): A transwell assay was used to analyze the migration ability in CDK12-inhibited TPC-1 and KAT-5 cell lines. Representative images of migrated cells are shown in the left panel, and the calculated results are shown in the right panel. (B): Metastasis *in vivo* model: representative IHC images of metastatic nodule sections are shown in the left panel, and thequantity of lung nodules is summarized in the right panel. **, *P*<0.01.

Cancer cell migration is vital intumor metastasis. Strong migration ability can accelerate the occurrence of metastasis. Therefore, we conducted a transwell assay and metastasis assay *in vivo* to study the mechanism of CDK12 in cancer metastasis. The transwell results showed that reduced CDK12 expression resulted in weaker migration ability compared with that of the control group (Figure 3A). The animal metastasis model also verified that the lack of CDK12 inhibited cancer metastasis, and the metastatic lung nodule loads were lower than those in the sh-CTR group (Figure 3B). In conclusion, CDK12 promotes cancer metastasis *in vivo* and *in vitro*.

### CDK12 promotes tumor progression by regulating c-myc/β-catenin pathway activation

**Figure 4:**
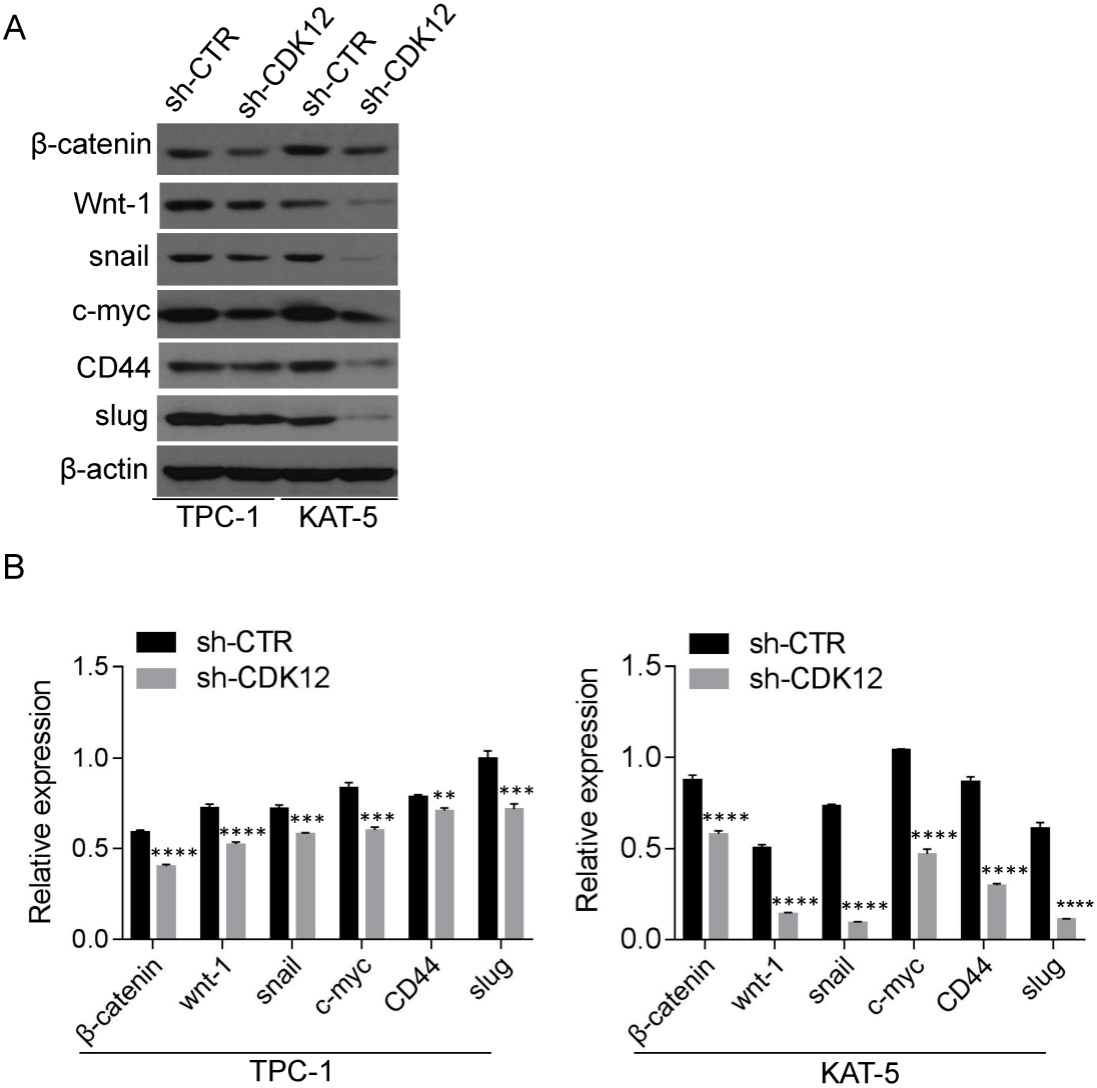
CDK12 activates the c-myc/b-catenin pathway and may regulate cancer cell stemness (A): Western blotting was utilized to assess the relative expression of wnt-1, c-myc, CD44, β-catenin, slug and snail in TPC-1-sh-ctr and KAT-5-sh-CDK12 cells. Actin was used as the internal control. **, P<0.01; ***, P<0.001;****, P<0.0001.(B): the quantitative analysis of the Western blot, **, P<0.01; ***, P<0.001;****, P<0.0001.

The c-myc/β-catenin pathway is a classical signaling pathway in cancer pathogenesis and is involved in cell stemness maintenance. Slug and snail were reported downstream of β-catenin and c-myc. The Western blot results revealed that CDK12 is closely related to the c-myc/β-catenin pathway (Figure 4). The proteins in the Wnt pathway, such as Wnt-1, β-catenin, c-myc, snail, and slug, were downregulated when CDK12 was knocked down. As a well-known stem cell marker, the expression of CD44 also decreased when CDK12 expression was inhibited. This indicates that CDK12 plays a vital role in the stemness of PTC cells.

## Discussion

Although papillary thyroid cancer generally has a good prognosis, the outcome of papillary thyroid cancer metastasis is still unsatisfactory 27. Few effective therapies can be applied for papillary thyroid cancer metastasis.

CDK12 acts as a cyclin-dependent kinase (CDK) that assembles with cyclin K to play a key role in cell cycle transition 28. CDK12 maintains genomic stability byrepairing DNA damage. The loss of CDK12 can lead to carcinogenesis 29. In this paper, we explored whether CDK12 is upregulated in PTC clinical specimens. The CDK12 expression in PTC cell lines further confirmed the association between CDK12 and PTC carcinogenesis. Our data demonstrated that inhibiting CDK12 expression *in vivo* and *in vitro* significantly suppressed cancer cell proliferation and tumor weights.

Metastasis is a vital challenge in cancer therapy, and it enormously threatens patient lives. Decreasing the occurrence of metastasis can effectively prolong patient survival time and reduce suffering 30. The transwell and metastasis assay results showed that CDK12 reduced cancer cell migration and the number of lung metastatic nodules. These results indicate that CDK12 can be a potential target in PTC therapy.

The c-myc/β-catenin pathway is an important pathway in tumorigenesis. The major Wnt pathway signals depend on β-catenin. Wnt activates the receptors on the membrane surface. Then, β-catenin is activated in the cytoplasm and transferred into the nucleus. β-catenin in the nucleus regulates the expression of target genes, such as c-myc 31. Slug and Snail are the downstream targets of c-myc. Slug and Snail regulate cell adhesion ability and play important roles in the epithelial-to-mesenchymal transition (EMT) 32-34. Through a silicon assay, we found that CDK12 participated in the c-myc/β-catenin pathway to promote tumor progression. Western blotting confirmed that CDK12 participates in the wnt pathway to promote PTC and EMT progression. Our results help to complete the map of the c-myc/β-catenin pathway and provide a new potential target for PTC therapy.

CD44 is a well-known stemness marker. Cancer cell stemness is regarded as the cause of cancer relapse. CSCs can self-renew and maintain the ability to differentiate into other cancer types that display higher drug-resistance and malignant features 35. The decreased expression of CD44 resulting from CDK12 inhibition revealed that CDK12 may affect the stemness of PTC cells.

In summary, CDK12 can inhibit cancer progression and metastasis by affecting cell proliferation and migration and inhibiting c-myc/β-catenin pathway expression and cancer stemness. CDK12 might become a potential biomarker or therapy target of PTC.

## Conclusions

CDK12 can affect the c-myc/β-catenin pathway to stimulate papillary thyroid cancer proliferation and metastasis.CDK12 might be a new therapeutic target for papillary thyroid cancer.

## Supplementary Material

Supplementary table.
