# Supplementary material for: CDK12 promotes papillary thyroid cancer progression through regulating the c-myc/β-catenin pathway
Source: J Cancer. 2020 Apr 27;11(15):4308–15. doi: 10.7150/jca.42849 (PMC7255385; doi:10.7150/jca.42849)
Supplement: Supplementary file 1 — Supplementary table. [file jcav11p4308s1.pdf]

supplemental material

Table1 the clinical characteristic of PTC patients

Variable	CDK12 expression			*P value*
	Case	High	Low	
Ages(Years)				0.904
<50	16	6	10	
>50	14	6	8	
Tumor size				0.018
<2.0 cm	19	13	6	
>2.0 cm	11	8	3	
Lymph node status				0.506
negative	21	9	12	
positive	9	4	5	
TNM stage				0.021
I-II	23	10	13	
III-IV	7	5	2	
